# Organizational HIV monitoring and evaluation capacity rapid needs assessment: the case of Kenya

**DOI:** 10.11604/pamj.2013.14.129.2581

**Published:** 2013-04-03

**Authors:** Mwende Mbondo, Jennifer Scherer, Gilbert Onyango Aluoch, Aaron Sundsmo, Njeri Mwaura

**Affiliations:** 1 PSI Kenya, Jumuia Place, Lenana Road, Box 22591–00400, Nairobi, Kenya, 254–20–2714342 (facsimile); 2 Danya International, Inc., 8737 Colesville Road, Suite 1100, Silver Spring, Maryland 20910, United States of America, (301) 565–3710 (facsimile); 3 Danya International, Inc., Nivina Towers, First Floor, Westlands Road, P.O. Box 14259–00800, Westlands, Nairobi, Kenya; 4 AMREF in Kenya Country Office, Wilson Airport, Lang'ata Road, Nairobi, Kenya

**Keywords:** HIV/AIDS, monitoring and evaluation, strategic information, rapid needs assessment, Kenya, M&E capacity building, Kenya National HIV/AIDS Strategic Plan (KNASP), Centers for Disease Control and Prevention

## Abstract

**Introduction:**

Due to the commitment by the Government of Kenya (GoK) and international donors to address HIV/AIDS, Kenya has some of Africa's most developed health infrastructure for tackling the crisis. Despite this commitment, significant gaps exist in the national HIV/AIDS monitoring and evaluation (M&E) system. To identify these gaps and opportunities for improvement, the U.S. Centers for Disease Control and Prevention funded the Strengthening HIV Strategic Information in Kenya project, which conducted an organizational HIV M&E capacity rapid needs assessment (RNA).

**Methods:**

The project included an in-depth desk review of national documents, policies, tools, and international best practices. National, regional, and district officials from government agencies, development partners, and implementing partners participated in key informant interviews and focus group discussions. Given the large number of regions and districts, purposive sampling was used to select 16 facilities in 8 districts across 2 regions based on the general quality of the reported HIV data and the number of partners supporting the regions.

**Results:**

RNA findings revealed tremendous improvements at the national level and in the various subsystems that contribute to the overall HIV strategic information. There also were significant gaps, including in a lack of M&E guidelines, parallel reporting systems, feedback given to subnational levels, and data use and general data management and use capacity at subnational levels.

**Conclusion:**

An urgent need exists for the development of national M&E guidelines and a comprehensive training curriculum. To ensure success further, capacity building for subnational levels should be conducted and feedback channels to subnational staff should be established and maintained.

## Introduction

The management of HIV and AIDS implementation activities in Kenya, through coordination of the National AIDS Control Council (NACC), is guided by 5-year national strategic plans, the current one being the Kenya National AIDS Strategic Plan III (KNASP III) for the period 2009/10-2012/13 [1]. While the implementation of successive strategic plans has been generally successful, some challenges still persist, especially in regard to timely HIV data and the availability of current information for decision making.

The U.S. Government's President's Emergency Plan for AIDS Relief (PEPFAR) funds several projects in Kenya to address some of the strategic information needs for HIV and AIDS programming. One such effort is led by ADAM, a consortium of four partner organizations - the African Medical and Research Foundation (prime), Danya International, AfriAfya, and ICF Macro - jointly implementing the Strengthening HIV Strategic Information in Kenya project, which is funded by the U.S. Centers for Disease Control and Prevention (CDC). The overarching goal is “to strengthen the capacity in Kenya to collect, manage, and use HIV/AIDS data and information at the national and decentralized levels” as a contribution to KNASP III's “one agreed country-level (monitoring and evaluation, M&E) system.” [2].

ADAM's project implementation team (PIT), in collaboration with ICF's Division of Health Information Systems (HIS), NACC, and the National AIDS and STI Control Program (NASCOP), conducted a participatory M&E capacity rapid needs assessment (RNA) to collect baseline data to guide the project's implementation. The RNA addressed 6 of the 12 components of a functional national HIV M&E system as endorsed by the Joint United Nations Programme on HIV/AIDS (UNAIDS) [3], and was based on ADAM's contractual mandate. Specifically, the RNA addressed organizational structures, human capacity, partnerships, routine program monitoring, supportive supervision and data auditing, and data dissemination and use. The assessment, conducted between February and May 2010, targeted the Western province, the North Rift Valley region, and institutions at the national level in Nairobi.

## Methods

The assessment involved conducting a desk review, key informant interviews, focus group discussions (FGDs), and an M&E stakeholders’ forum. The desk review's purposes were to provide the assessment a starting point through articulation of the international M&E standards that would guide the activity, and to review the various HIV M&E documents, including data sets and reporting tools. The PIT modified the M&E system strengthening tool that assesses the UNAIDS-endorsed organizing framework for a functional national M&E system to address 6 of the 12 components that were in-line with the project's mandate for this study. Once NASCOP approved the revised tool, the PIT used it to conduct key informant interviews and FGDs.

**Figure 1:**
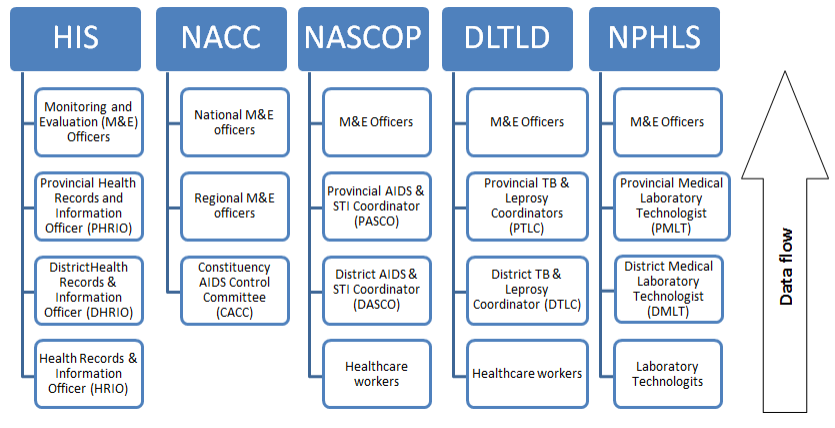
Organizational structure of the different Government of Kenya health agencies

The PIT conducted structured key informant interviews with national-level government, development, and implementing partners, and FGDs with the Government of Kenya (GoK) provincial, district, and health facility staff in 2 of the 10 NASCOP regions (Figure 1). The PIT, in consultation with NASCOP, selected the two regions based on the overall quality of HIV data and the number of implementing partners working in the region. Within these two regions, the PIT employed purposive sampling to select four districts, based on their number of comprehensive care centers (CCCs). The PIT selected two facilities from each of the selected districts, according to the following criteria: health facilities that have CCCs or are providing antiretroviral therapy; and GoK and private facilities. The PIT further refined the regional participants in the field through consultation with the provincial health records and information officers (PHRIOs).

The RNA addressed several key questions, including: What are the strengths, capacity gaps, and challenges in M&E of HIV and AIDS interventions in Division of Leprosy, Tuberculosis, and Lung Disease (DLTLD), HIS, NACC, NASCOP, and National Public Health Laboratory Service (NPHLS) systems; what are the strengths and challenges among the development and implementing partners in aligning with the national M&E system; and what are the possible capacity-building interventions that can be applied to meet the identified challenges and gaps?

The PIT conducted a total of 28 key informant interviews, including 11 from GoK and 17 from development and implementing partner organizations, and 19 FGDs. Table 1 and Table 2 depict the number of participants interviewed and those who participated in the FGDs, respectively.

**Table 1:** Number of key informants interviewed during the rapid needs assessment (RNA)

Key informants interviews	Number of key informants	Number of organizations
Government of Kenya	11	5
Developing partners	3	3
Implementing partners	14	9
**Total**	**28**	**17**

**Table 2:** Number of focus group discussions (FGDs) and participants in each region during the RNA

FGDs	Number of FGDs		Total number of participants	
	Western	North Rift	Western	North Rift
Provincial health management team	1	2	11	16
District health management team	2	3	17	22
Constituency AIDS control committees	1	2	13	24
Data officers	2	2	16	21
Facility health workers	2	2	18	13
**Total**	**8**	**11**	**75**	**96**

Once the PIT collected the information, it convened data interpretation sessions with national-level staff from DLTLD, HIS, NACC, NASCOP, and NPHLS. The purpose of these participatory data interpretation sessions was to ensure complete understanding and ownership of the data, thereby leading to agreement on the way forward for implementation. The PIT disseminated the draft report from the sessions at a national forum where representatives from the provincial, district, facility, and community levels participated.

## Results

### Organizational structures and HIV M&E functions

The organizational structures of DLTLD, HIS, and NPHLS are divided into four levels: national/central, provincial, district, and health facility. However, NACC has a three-level structure consisting of national, provincial/regional, and constituency levels. Data are collected and collated at the lowest level, which is the health facility level for most government agencies and the constituency level for NACC. At each level, the PIT conducted basic data analysis before passing the data to the next level, and also collated all data from DLTLD, NACC, NASCOP, and NPHLS at the district level and provided them to the district health records and information officer (DHRIO) for reporting through HIS.

### Routine HIV program monitoring

NACC's community-based program activity report is the standardized tool for nongovernmental organizations, community-based organizations, AIDS control units, and private sector organizations to use when reporting HIV and AIDS activities in which they are involved. These organizations submit their reports to constituency AIDS control committees (CACCs), which collate the constituency data and forward them to the regional teams to be collated again and sent to the national level. The PIT discovered that CACCs need to be retrained on NACC policies, equipped with laptops to assist with coordination, and provided with improved linkages between NACC structures and stakeholders.

The PIT learned that NASCOP developed standardized tools for use in different HIV programs in public and private institutions, because some sections of the Antiretroviral Therapy and Provider-Initiated Counseling and Testing Register and the tuberculosis (TB) section of the MOH 711-integrated reporting tool were either difficult to complete or understand. The PIT also discovered that TB data reported through MOH 711 and reported by DLTLD captured the same variable yet results differed in most cases. In some regions, implementing partners developed their own tools due to the unavailability of the recommended standard tools.

The DHRIO used electronic File Transfer Protocol (FTP) to collate health facility data at the district level before submitting it to the provincial level. The DHRIO reported that using FTP simplified reporting processes, but lack of adequate FTP training for all personnel limited its use. The PIT determined that further training was needed on FTP use and protocols, basic information communication and technology (ICT), and data analysis and use. The PIT also noted that personnel needed clear guidelines on data management.

### Human capacity for HIV M&E

The PIT determined that the split of the health ministry into the Ministry of Public Health and Sanitation and the Ministry of Medical Services has resulted in lack of clarity, with overlaps in the roles and responsibilities of personnel at the district level. The PIT also noted a shortage of HRIOs, ICT officers, and statisticians within HIS at the decentralized level. At HIS, most staff had basic data capture and reporting skills, but had limited ability to check data quality, undertake basic data analysis and interpretation, and use the data generated for decision making. National-level staff at NPHLS had master's level educations in epidemiology, biostatistics, and information technology, but district- and health facility-level staff lacked adequate capacity.

### Issues affecting M&E capacity

NASCOP respondents viewed the lack of a nationally recognized M&E curriculum as an issue affecting M&E capacity. Additionally, NPHLS staff viewed the lack of electronic data management systems as a capacity gap.

### Identified capacity-building needs

HIS noted it needed to develop a capacity-building plan. NACC identified its needs, as a still relatively young organization, for continuous mentorship and on-job-training on M&E and ICT. NASCOP pointed to needs for training on: geographic information systems mapping for spatial data presentation, preparing and submitting abstracts and manuscripts, conducting operational research, and utilizing research data.

### Supportive supervision and data quality

According to its RNA responses, DLTLD conducts weekly supportive supervisory visits for urban centers and “problem centers”, monthly visits for diagnostic facilities, and quarterly visits for less busy treatment-only centers. The organization noted that it planned all supportive supervision visits on scheduled clinic days and provided supportive supervision to each level in the DLTLD structure. District TB and leprosy coordinators (DTLCs) conduct health facility-level supportive supervision, sometimes assisted by provincial TB and leprosy coordinators, who also conduct district-level supervision.

NASCOP plans supportive supervisions for every quarter, but often does not implement these plans due to funding limitations and competing tasks. NPHLS reported that its supportive supervision, which falls under the district health management team (DHMT), was hampered by lack of funding as well as less time being attributed to laboratory services during supportive supervision.

### Supportive supervision and data quality assessment tools

In some regions, HIS received support from implementing partners to develop supportive supervision tools. However, the PIT recommends that the tools be standardized and specific guidelines be developed to guide supportive supervision as well as data quality assessment (DQA) activities. For example, NASCOP reported how it developed a standardized supportive supervision tool with financial support from UNICEF. Interviews with NPHLS revealed that the organization had plans underway to revise DHMT's tool for supportive supervision to incorporate the laboratory system and M&E component.

### Dissemination of supportive supervision and DQA findings

NASCOP disseminated results from supportive supervision and DQA to provincial and relevant district personnel. However, the organization reported that it had no standard format for writing a supportive supervision report and lacked a strong dissemination plan for the final report.

### Data dissemination and use

HIS staff indicated during a discussion that data dissemination and use was perceived as “a matter of clearing and forwarding exercise”, thus, minimal effort occurred at the health facility level. In Western province, RNA respondents noted they were encouraged to initiate data sharing within district health facilities in the region. However, respondents indicated this process needs to be structured better to promote sharing and use of data.

NACC used the joint annual program review (JAPR) to review and share data among various stakeholders. JAPR brings together government line ministries and development and implementing partners to review their KNASP progress. The process begins with district and regional meetings that culminate in the national JAPR. PEPFAR partners benefit from data sharing during the semiannual program review and annual program review. Although the meetings primarily share data from PEPFAR-supported partners, HIS, NACC, and NASCOP representatives also participate. NACC reported that constituency-level data use for decision making was minimal.

### Partnerships to plan, coordinate, and manage the HIV M&E system

RNA respondents reported partnerships among GoK and development and implementing partners at all levels of the M&E system. At the national level, NACC and NASCOP serve as chair and co-chair, respectively, for an M&E technical working group (TWG), which includes representation from developing and implementing partners. TWG membership is fluid and determined based on the agenda items or issues on which to deliberate. Past TWGs have drawn participation from development partners such as CDC, the U.S. Agency for International Development, and the World Health Organization; government agencies such as Kenya Medical Research Institute and Kenya National Bureau of Statistics; and implementing partners such as Family Health International. RNA respondents indicated that TWGs are held on a quarterly basis.

Respondents also indicated that the main challenges of the TWGs were poor coordination among stakeholders, limited follow-up on meeting action points, lack of commitment by some stakeholders, inconsistent attendance, and lack of motivation to attend the meetings. RNA responses indicated that the government needed to provide greater TWG leadership to ensure a coordinated response to HIV and AIDS.

## Discussion

### Statement of the principal findings

In examining the information gathered through the organizational RNA, it is clear that major achievements occurred within the national HIV M&E sector. In totality, the National Health Sector Strategic Plan II review revealed that GoK, through the Ministry of Public Health and Sanitation and the Ministry of Medical Services as well as the Ministry of Finance, is making strides to ensure that appropriate indicators are captured to measure the HIV response. While this report presented the preceding findings according to each of the HIV M&E subsystems, the discussion that follows attempts to bring together all the key findings for each level.

### HIV M&E systems strengths

**National level**. The primary strength of the national HIV M&E system is the existence of a clearly articulated national strategy for HIV M&E alignment coordinated by NACC. Further, because it aligns with the UNAIDS-endorsed “Three Ones” principles, the KNASP III has one national M&E system that is identified under the KNASP III M&E framework. NACC, as the national coordinating entity for HIV and AIDS information, established a national M&E TWG that has representatives from a wide sector of HIV program implementing and development organizations in Kenya. NACC and NASCOP co-chair the national M&E TWG, providing a concerted effort toward the M&E activities for HIV in the health sector. The meetings provide the forum for fostering partnerships among the stakeholders at both the national and decentralized levels. Further, the presence of JAPR activities throughout the national and decentralized levels ensure that HIV and AIDS programs are reviewed at all levels, culminating in a national report that gives a clear picture of HIV and AIDS programming throughout Kenya. Within the national HIS, the streamlining of data through FTP is commendable. Having a national electronic reporting system for health data, including HIV and AIDS data, has been a remarkable accomplishment. As has been noted in the findings of this RNA, the implementation of FTP has greatly improved the reporting rates of HIV data at the national level.

**Provincial, district, and facility levels**. The most prominent strength of the HIV M&E system at the provincial level is the presence of adequate personnel from all the subsystems. This has resulted in significant representation on the provincial health management teams (PHMTs). At the district level, the most important strength is the ability to coordinate the health activities. Currently, most of the original districts have a DHMT with complete representation.

### HIV M&E gaps and challenges

**National level**. Despite having a national M&E framework that supports the KNASP III, the implementation of the M&E framework has faced challenges. While many partners work to support the various activities, they lack the ability to coordinate these activities. One of the challenges of each program having its own reporting system, from the facility to the national level, is that this has led to the creation of parallel systems with very little data sharing, even at the national level. Thus, opportunities for creating synergies in data collection processes and program implementation are sometimes missed, often leading to duplicate efforts in addressing HIV and AIDS issues. As documented in the desk review and field activities, there is scant coordination among DLTLD, HIS, NACC, and NASCOP. While these organizations are making efforts toward streamlining this process at the national level through the inclusion of representatives from all divisions, departments, and programs, their efforts have yet to have an impact at the decentralized levels.

Another key area that requires attention is the lack of written guidelines and/or standard operating procedures (SOPs) regarding data validation and routine data quality audits. Written guidelines on data validation assist the health records personnel at the facility, district, provincial, and national levels to provide accurate information for policy formulation and to inform program improvement. While there are many implementing partners working with HIS, NACC, and NASCOP, they do not have uniform standards of engagement, even with implementing partners affiliated with the same government division. The tools for HIV programs change frequently, which makes it difficult for the healthcare providers to stay abreast of any shifts. Compounded by the absence of tools and training on the updated tools, the providers encounter difficulty delivering accurate information on the HIV activities that they conduct in their facilities.

**Provincial level**. One of the biggest challenges at the provincial level is the lack of coordination among all the various subsystems and implementing partners. The PHMT holds regular meetings, but it focuses minimally on data. While partners do sometimes support forums for sharing HIV information, they are not well coordinated and data are hardly ever discussed.

**District and facility levels**. The district and facility levels lie at the heart of program implementation and, therefore, serve as the custodians of a wealth of information. However, several factors contribute to the challenges of managing this information. First, the number of districts is increasing faster than an appropriate number of staff can be located and hired. Within the national health sector in Kenya, an acute staff shortage exists at the facility and district levels. RNA findings highlight this fact, which has a major impact on the functioning of the national HIV M&E system. The organizing framework for a functional national HIV M&E system [2] describes the 12 components of a functional HIV M&E system and places emphasis, not only on having staff dedicated for M&E at all levels of the system, but also on having staff with the adequate training to conduct their activities. At the same time, there has been minimal comprehensive training as well as refresher training provided on updates made on the data collection tools.

Second, the numerous tools, including those required by the various implementing partners, add to the healthcare providers’ workloads. Training notwithstanding, healthcare providers have an insufficient understanding of data, especially those providers at the facility level. This is aggravated by the fact that there is no feedback on the HIV data submitted from the health facility to the national level. Similarly, while organizations conduct supervision visits with the intention of monitoring the activities at the peripheral levels, they rarely ever provide even minimal written feedback. Thus, no documentation exists to ensure that required follow-up occurs. The lack of HIV data sharing among stakeholders from the facility through the national level, as noted in the assessment, can lead to incomplete data and poor understanding of the true picture of HIV in the country at all levels. It also means that decisions are made on HIV programming that may not be beneficial toward attaining the objectives outlined in the KNASP III.

### Strengths and weaknesses of the study and in relation to other studies

The greatest strength of this study lies in the comprehensive and transparent engagement of key stakeholders throughout the process (not just at the outset). The study began with the identification and mapping of potential stakeholders. The PIT strove to be more inclusive than exclusive because it did not want to unintentionally create any feelings of alienation. Upon identification of the final set of key stakeholders, the PIT recognized that relationship building with stakeholders takes time, so it establish a long-term proactive communication plan defining how it would interact with and inform key stakeholders. Establishing communication channels provided a mechanism for clear and consistent information flow between the project team and the stakeholders. Good communication led to more trusting relationships and camaraderie among the stakeholders. The PIT continues to engage the key stakeholders in the remaining project activities such as the development of the M&E guidelines and the development of the curriculum and training materials. The benefits of constructive stakeholder engagement were significant, including better end products and outcomes.

The PIT understands that limitations to this study exist. Given that this work targeted only some components of the UNAIDS-endorsed 12 components of a functional M&E system, the results of the exercise only reflect those components that were reviewed. The selection of the regions for the RNA field activities, while done in consultation with NASCOP, may not be a true reflection of the M&E strengths, challenges, gaps, and opportunities for improvement in the country as a whole. These regions may have their own unique factors that may or may not be found in other parts of Kenya. In addition, the views expressed by those interviewed during this exercise are based on their experience working in their specific regions. The involvement of national-level participants from all the subsystems helped provide a national outlook that was meant to give insight on systematic issues of the subsystems.

The authors did not locate any highly similar published studies conducted in Kenya and, thus, are unable to compare this work to that of others.

### Future research

Given the mandate of improving the HIV M&E system, the PIT provides the following recommendations in order of importance on the areas where the improvements can provide the greatest support:

1. **Develop M&E guidelines and SOPs for NASCOP** - To address the issue of lack of standards for HIV data, there is a need to develop national HIV M&E guidelines and SOPs that can be used throughout the country. These guidelines should include information on how to: conduct M&E supervision and data quality audits, work with implementing partners, and establish roles and responsibilities for all stakeholders.
2. **Develop standard M&E training curriculum** - To ensure that all healthcare workers are updated on various aspects of M&E, a standard curriculum for training on M&E should be developed, which will assist in availing M&E courses to the staff at minimal cost. The curriculum must be comprehensive and target all levels of health workers, with trainers instructed to roll it out throughout Kenya.
3. **Provide forums for sharing data on a regular basis** - Regular forums for sharing data at all levels should occur at least on a quarterly basis. This includes the forums for sharing data among HIS, NACC, and NASCOP as well as all other HIV stakeholders, including implementing and development partners.
4. **Focus M&E strengthening on the facility and district staff** - While strengthening HIS to be able to carry out its core functions as far as data are concerned, it also is recommended that the healthcare personnel at the facility and district levels be the focus of a major part of the system strengthening. Training on the national tools should be provided to the actual data collectors at the facility level, initially at a central venue, but with follow-up at the facility level to address any issues that may arise.
5. **Strengthen HIS's ability to collect, analyze, and interpret data** - As the governmental division charged with the collection, analysis, and reporting of all health information in the country, HIS needs to be strengthened to be able to carry out its mandate. This requires additional qualified staff, including biostatisticians and epidemiologists, and also the capacity building of the staff currently working at the division.

## Conclusion

RNA results indicate a strong need for the development of national M&E guidelines and training curricula. To retain the newly acquired technical skills and knowledge, capacity building for subnational levels must occur and organizations must establish and maintain feedback channels to the subnational staff to encourage the multi-channel flow of communication.
